# Comparative study of initial stages of copper immersion deposition on bulk and porous silicon

**DOI:** 10.1186/1556-276X-8-85

**Published:** 2013-02-15

**Authors:** Hanna Bandarenka, Sergey L Prischepa, Rosalba Fittipaldi, Antonio Vecchione, Paolo Nenzi, Marco Balucani, Vitaly Bondarenko

**Affiliations:** 1 Department of Micro- and Nanoelectronics, Belarusian State University of Informatics and Radioelectronics, 6 Brovka St., 220013, Minsk, Belarus; 2 Department of Telecommunications, Belarusian State University of Informatics and Radioelectronics, 6 Brovka St., 220013, Minsk, Belarus; 3 CNR-SPIN Salerno and Dipartimento di Fisica ‘E.R. Caianiello’, Università degli Studi di Salerno, I-84084, Fisciano, Salerno, Italy; 4 Department of Information Engineering, Electronics and Telecommunications, University ‘Sapienza’, 18 Eudossiana St., 00184, Rome, Italy

**Keywords:** Porous silicon, Copper nanoparticles, Immersion deposition, Electron backscatter diffraction

## Abstract

Initial stages of Cu immersion deposition in the presence of hydrofluoric acid on bulk and porous silicon were studied. Cu was found to deposit both on bulk and porous silicon as a layer of nanoparticles which grew according to the Volmer-Weber mechanism. It was revealed that at the initial stages of immersion deposition, Cu nanoparticles consisted of crystals with a maximum size of 10 nm and inherited the orientation of the original silicon substrate. Deposited Cu nanoparticles were found to be partially oxidized to Cu_2_O while CuO was not detected for all samples. In contrast to porous silicon, the crystal orientation of the original silicon substrate significantly affected the sizes, density, and oxidation level of Cu nanoparticles deposited on bulk silicon.

## Background

Electrochemical anodizing of bulk crystalline silicon (Si) at specific conditions causes the formation of chaotic or ordered pore channels in its volume [1]. The material formed by such artificial nanostructuring is called porous silicon (PS). This porous morphological type of silicon presents an object of great interest of the scientific community because, in contrast to the bulk silicon, it demonstrates a number of peculiarities such as extremely developed surface, photo- and electroluminescence, and biocompatibility. Possession of these properties makes PS applicable to the areas of optoelectronics and display technologies, micromechanical systems, biomedicine, etc. The challenge to develop and engineer novel devices and technologies based on PS forces researchers to actively seek methods to control and manage the PS properties. One way to realize it is the incorporation of metal nanoparticles (NPs) into the pores of PS by deposition from wet solutions. Unlike dry methods (evaporation or sputtering), wet deposition provides deep penetration of metal atoms into pore channels [2]. Moreover, wet technologies are characterized by simplicity and low cost.

Immersion deposition presents a less complicated wet method of PS metallization. In contrast to electrochemical and chemical depositions, in this process, a source of the electrons for metal atoms reduction is PS itself. In aqueous solutions, the ions of metals, which have redox potential greater than hydrogen, attract electrons from Si atoms and are reduced to the atomic form [3]. The immersion deposition of other metals can be carried out by the use of alkaline solutions [4]. During wet deposition, metal structures tend to grow as island films according to the Volmer-Weber mechanism [5]. Penetration of metals into PS may be easily controlled by the alternation of PS porosity [6]. Therefore, it is possible to fabricate metal films on the outer surface of PS or metal/PS nanocomposites (NCs). Obviously, during the immersion process, the Si skeleton of PS is oxidized, and SiO_2_ is formed under deposited metal structures [3,7]. The oxide's interlayer prevents further redox reactions between Si and metal ions, and as a result, there reduction of metal stops. Usually, to avoid the effect of oxidation, immersion deposition in the presence of fluoride species is performed [8,9]. In this case, SiO_2_ removal followed by Si oxidation caused the dissolution of the PS skeleton. Proper conditions of the metal immersion deposition and PS parameters can lead to the complete conversion of PS to porous metal [10].

The structures formed by immersion deposition of metals on PS are widely studied to be successfully applied in some technologically important areas [11-15].

Recently, it has been found that tensile strength of NCs formed by short copper (Cu) immersion deposition in PS is controlled by the variation of PS porosity [11]. Further research in this direction has shown that the sacrificial Cu/PS NCs have an opportunity to be successfully applied for the layer transfer in MEMS technology [12]. The presence of Cu NPs on the pore walls of PS promotes electrochemical deposition of thick metal films (the maximum thickness of metal film without the interlayer of Cu/PS NC is less than 2 μm). Moreover, electrochemical deposition of metals on p-type Si requires high potential value which compensates the lack of electrons for charge transfer. In case of PS formed on p-type Si and covered with Cu NPs, there is no need to apply a specific potential regime.

PS covered with silver (Ag) NPs by immersion deposition has been declared as an active substrate for the application in surface-enhanced Raman spectroscopy [13]. The enhancement factor of Ag/PS was evaluated to be about 10^8^ in comparison with that of substrates formed by immersion deposition of Ag on bulk Si under the same conditions. The authors reported that the developed surface of PS provides better covering with Ag NPs in contrast to bulk Si due to a greater number of active places. However, comparative quantification of Ag immersion deposition on bulk Si and PS has not been performed.

Porous Cu film fabricated by immersion technique from PS has been reported to demonstrate usability as a flexible electrode for electroporation [13]. The electrode presents a porous Cu membrane on the polymer substrate which is wrapped around the living tissue. Simulations have shown that the treated depth of tissue during the pulsed regime of electroporation reaches the value of 1 cm. The most significant advantages of such porous Cu films are flexibility, mechanical strength, and good adhesion to the polymer substrate [13,16].

Moreover, NCs and porous metal films formed by immersion deposition of metals in PS are prospective materials for the electrodes of Li-ion batteries, supercapacitors, and catalytic membranes of fuel cells [14,15].

The successful application of materials formed by immersion deposition of metals on PS strongly depends on technology repeatability. The development of such technology requires deep study of the properties of such materials at all stages of immersion deposition. The mechanisms of metal immersion deposition on PS as well as the properties of the final materials have been widely studied [17-19]. However, previous reports have presented the analysis of the initial stages of deposition in abbreviated form.

In the present work, we have reported the detailed study of immersion deposition of Cu on PS in comparison with bulk Si from aqueous solution of copper sulfate (CuSO_4_·5H_2_O) and HF. Initial understanding of the mechanism of Cu deposition was given by measurements of open-circuit potential (OCP) of Si and PS surfaces during immersion and scanning electron microscopy (SEM) of the experimental samples formed at different time periods. Principal attention was paid to the phase and structural analyses of Cu NPs which are formed at the initial stages of deposition. These NPs cannot be studied by means of X-ray diffraction (XRD) due to their extremely small sizes and trace amount. Such analysis was performed by electron backscatter diffraction (EBSD) which allowed scanning of the sample surface with a 2-nm resolution up to a 100-nm depth. It is necessary to note that the Cu lattice cell is similar to that of most metals usually deposited by immersion technique on bulk Si and PS (Ag, Ni, Au, Pd, and Pt). We suppose that the NPs of such metals grow on bulk Si and PS similarly with Cu NPs, and our findings are important to researchers with close interests in the metallization of PS by immersion deposition.

## Methods

Antimony-doped 100-mm monocrystalline silicon wafers of (100) and (111) orientations and 0.01-Ω·cm resistivity were used as initial substrates. Chemical cleaning of the Si wafers was performed for 10 min with a hot (75°C) solution of NH_4_OH, H_2_O_2_ and H_2_O mixed in a volume ratio of 1:1:4. After that, the wafers were rinsed in deionized water and dried by centrifugation. The wafers were then cut into a number of rectangular samples of 9 cm^2^ area. Some of samples were used to deposit copper on the surface of original bulk Si for comparative study with PS. Just before PS formation or immersion deposition of copper, each experimental sample was etched in 5% HF solution for 30 s to remove the native oxide. Immediately after oxide removal, the Si sample was placed in an electrolytic cell made of Teflon. The active opening of the cell had a round shape and an area of 3 cm^2^. Uniform PS layers were formed by electrochemical anodizing of silicon samples in a solution of HF (45%), H_2_O, and (СН_3_)_2_СНОН mixed in a 1:3:1 volume ratio. A spectrally pure graphite disk was used as contact electrode to the back side of the samples during the electrochemical treatment. Platinum spiral wire was used as cathode electrode. Anodizing was performed at a current density of 60 mA/cm^2^ for 20 s. After PS formation, the HF solution was removed, and the electrolytic cell was thoroughly rinsed in (СН_3_)_2_СНОН to remove products of the reactions from the pores.

To perform Cu deposition, we filled the cell containing Si or PS/Si samples with aqueous solution of 0.025 M CuSO_4_·5H_2_O and 0.005 M HF for different time periods. After that, the solution was poured out, and the cell was rinsed with (СН_3_)_2_СНОН. The sample was then taken of the cell and dried by flow of hot air at 40°C for 30 s.

OCP measurements were carried out using the Ag/AgCl reference electrode filled with saturated KCl solution. The reference electrode was immersed into a small bath filled with the solution for Cu deposition. The bath was connected to the electrolytic cell by a flexible polymer tube with a 2-mm inner diameter, with a Luggin glass capillary of 200-μm aperture at its end. Both the Luggin capillary and polymer tube were filled with the solution for Cu deposition. The Luggin capillary was placed on Si or PS, and it defined a clear small sensing point for the reference electrode near the sample surface.

The equipment used to conduct electrochemical processes was the AUTOLAB PGSTAT302n potentiostat/galvanostat (Utrecht, The Netherlands). The gravimetric method was applied to determine the porosity of PS and the mass of the deposited metal. Mass measurements were performed with a Sartorius CP225D micro/analytical electronic balance (Goettingen, Germany). The instrument mass error was 10 μg. The morphology of the samples was studied by SEM (Hitachi S-4800, Chiyoda-ku, Japan) with a resolution of 1 nm.

The analysis of the microstructure of the samples was performed with LEO EVO 50 scanning electron microscope (Carl Zeiss AG, Oberkochen, Germany) equipped with an Oxford Inca EBSD detector (Oxford Instruments plc, Oxfordshire, UK). Software from HKL Technology (Hobro, Denmark) was used for phase identification. An electron beam scanned the surface of the tilted sample placed in the SEM with a step size of 10 nm. The sample was steeply tilted to about 70° from the incident beam. EBSD measurements were performed at an electron high tension of 20 kV and probe current of 10 nA. A phosphor screen coupled to a Peltier cooled CCD camera was fluoresced by electrons from the sample to form the diffraction pattern [20].

## Results and discussion

In this work, our attention was paid to study the initial stages of Cu immersion deposition on PS consisting of ordered cylindrical pores which are perpendicularly oriented to the surface of the original Si substrate [21]. Exactly such kind of PS has been reported to be one of the most suitable host materials for the formation of NCs [22,23]. In particular, variation of the PS parameters without disordering pores allows the controlling of features of the final material. To understand peculiarities of Cu immersion deposition on the surface of PS, we firstly studied the process on the bulk Si because the surface of the PS pores presents Si nanoplanes of different crystal orientations [10]. Anodizing regimes used in this work provided formation of the uniform PS layers of 1-μm thick and 50% to 55% porosity. The diameter of pore channels and thickness of pore walls varied in the range from 10 to 50 nm, according to the evaluation of SEM images [9].

### Morphology of Cu/Si and Cu/PS/Si samples

**Figure 1:**
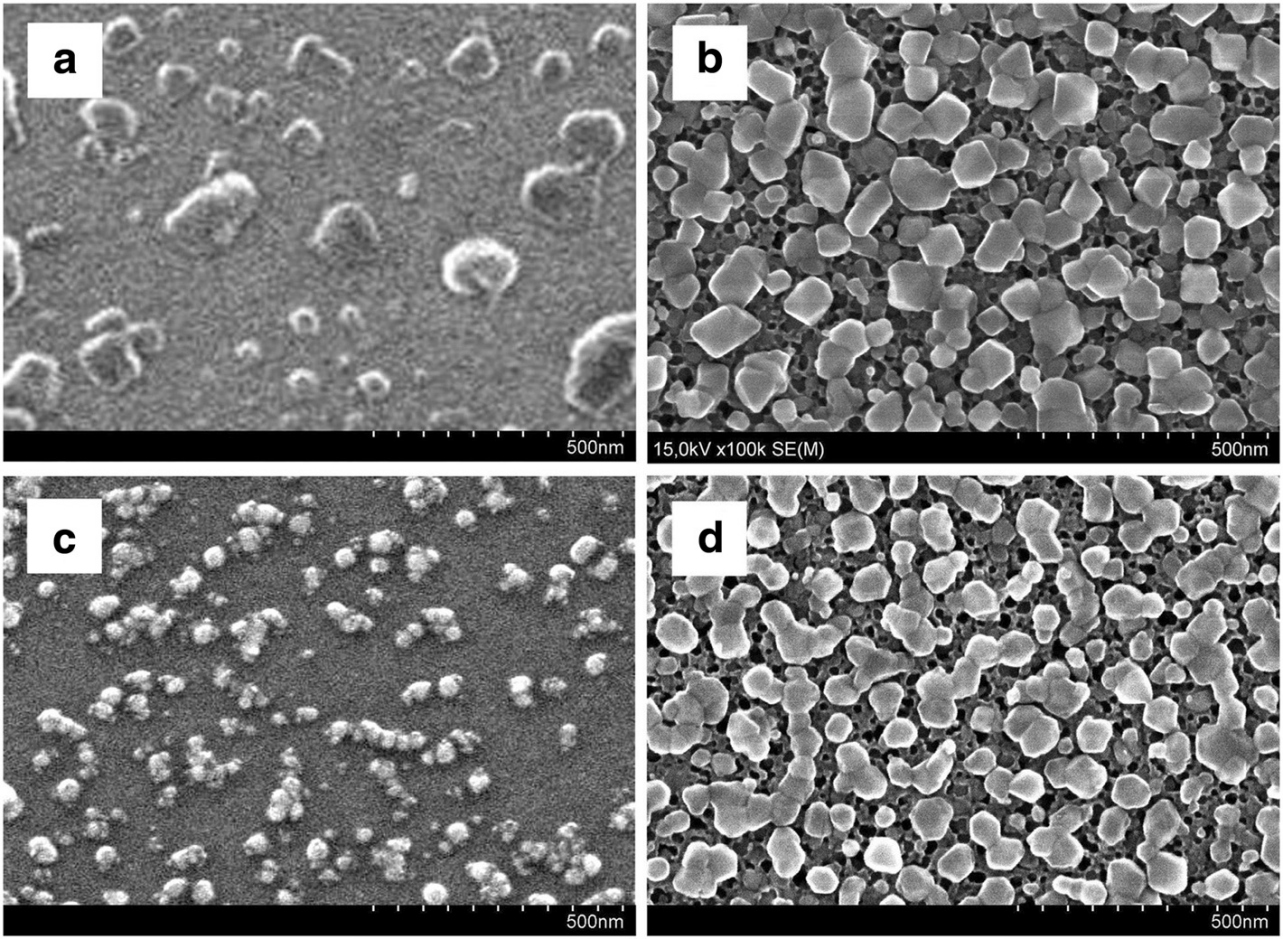
**SEM analysis of the surface of samples. **(**a**) Cu/Si (100), (**b**) Cu/PS/Si (100), (**c**) Cu/Si (111), and (**d**) Cu/PS/Si (111).

**Figure 2:**
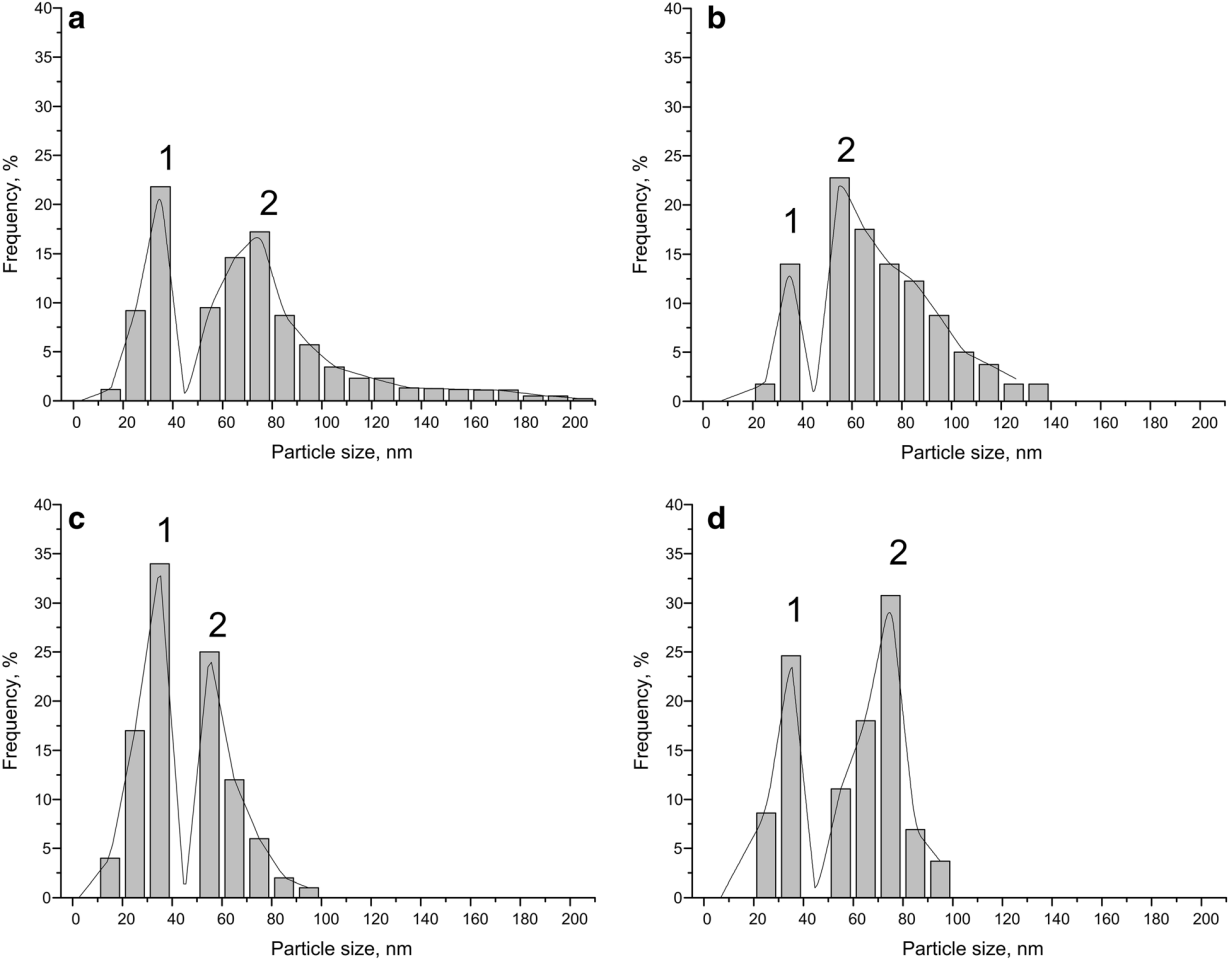
**Size distribution histograms.** Histograms were made by computer evaluation of SEM images presented on Figure 1. (**a**) Cu/Si (100), (**b**) Cu/PS/Si (100), (**c**) Cu/Si (111), and (**d**) Cu/PS/Si (111).

Figure 1 shows SEM views of the surface of the bulk Si (Figure 1a,c) and PS (Figure 1b,d) samples after immersion into the solution for Cu deposition for 4 s. Figure 1a,b corresponds to the substrates based on Si (100), while Figure 1c,d was formed on Si (111). It is well observed that Cu deposited on PS as a quasi-continuous film that consists of connected NPs (Figure 1b,d), while bulk Si was covered with the separated NPs (Figure 1a,c). Figure 2 presents the size distribution histograms of Cu particles based on the measurements of 100 particles from each SEM (Figure 1). The common characteristics of all histograms are bimodality of the distributions and absence of the particles in the range from 40 to 50 nm. The average diameters related to the first part of the size distributions were almost the same for all samples (30 to 35 nm), while in the second part, the average diameter for Si (100) was estimated to be 85 nm; for Si (111), 55 nm; and for both PS samples, 70 to 75 nm. Therefore, in such case, PS sizes of the Cu NPs were not affected by the original Si orientation in contrast to the bulk Si. Such bimodality of the histograms means that the initially deposited Cu NPs have already coalesced into larger particles (agglomerates) - the second part of the distributions - and new NPs deposited on the reopened surface of the substrates - the first part of the distributions. This mechanism usually takes place in wet depositions [5,10]. The density of Cu particles on the Si (100) estimated as 10^9^ cm^−2^ was an order of magnitude less than those on Si (111) and PS, which are 10^10^ and 2 × 10^10^ cm^−2^ (for the both orientations), respectively. Considering the less density and greater sizes of Cu particles on the bulk Si (100), we suppose that the orientation promotes faster coalescence of Cu NPs. Cu NPs have higher mobility due to less number of broken bonds on the Si (100) surface in contrast to Si (111). A greater number of Cu NPs on the PS samples in comparison with bulk Si shows that the porous surface provides more active places for Cu adhesion and nucleation.

### Microstructure of Cu/Si and Cu/PS/Si samples

**Figure 3:**
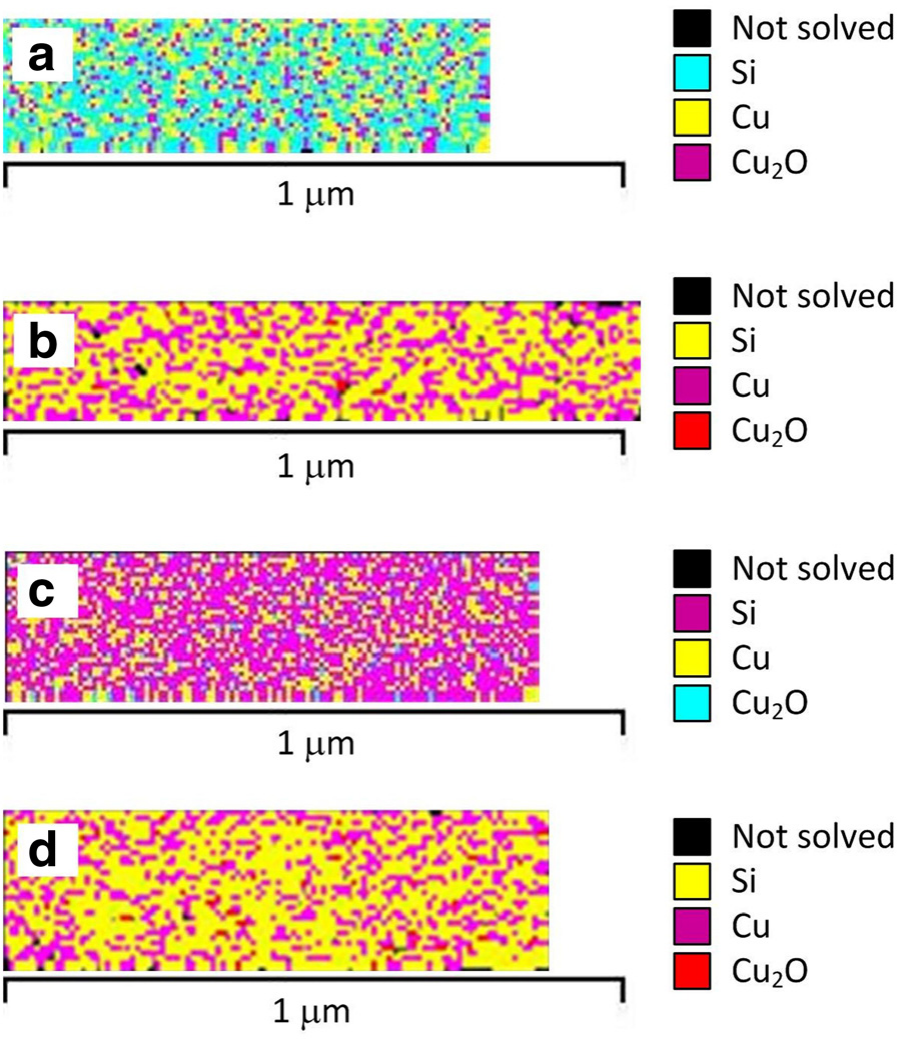
**EBSD phase maps.** Illustrations of phase discrimination were obtained for the surface region of samples (**a**) Cu/Si (100), (**b**) Cu/PS/Si (100), (**c**) Cu/Si (111), and (**d**) Cu/PS/Si (111).

XRD analysis of the phase composition and crystal orientation of PS after Cu immersion deposition has shown the presence of Cu, Cu_2_O, and rarely CuO crystalline phases in the deposit [24]. However, no data were obtained for the initial stages of the Cu immersion deposition because XRD is not sensitive to trace the amounts of crystals of small sizes. To solve the problem, we used EBSD which allows the local study of crystalline object microstructure. Before EBSD analysis, the crystallographic data of the Si, Cu, Cu_2_O, and CuO phases were entered into the customized HKL channel 5 software database for phase identification. Figure 3 presents the phase maps of the Si and PS surfaces after Cu immersion deposition for 4 s. Table 1 shows the quantitative data of the mapping which resulted in some disagreements with the SEM analysis. According to the phase maps, the Cu amount did not differ greatly for all samples, while the SEM images revealed significant variations of the Cu NP density. We explain it in the following way. The electron beam energy provided a 100-nm depth in the analysis. During EBSD scanning, the samples were tilted, so the electron beam penetrated under the Cu NPs or into the pores of PS, detecting internal Si crystals in the pore walls. That introduced an error in the phase distribution. Nevertheless, it is shown that films deposited by Cu immersion deposition on Si and PS are noncontinuous, have a crystalline nature, and consist of Cu and Cu_2_O crystals of the cubic lattice cell. CuO was not found. The step size of EBSD scanning was 10 nm, which means that crystals of such dimensions exist in the deposited films. It should be noticed that Cu NPs deposited on the bulk Si (100) are oxidized more (amount of Cu_2_O is 13%) than other samples (Table 1).

**Table 1:** Results of EBSD analysis of bulk Si and PS surfaces covered with Cu

**Sample type**	**Phase**	**Percentage (%)**	**Count**	**Area (mm**^**2**^**)**	**Orientation**	**Lattice cell**
Cu/Si (100)	Not detected	15.9	437	0.03	None	Unsolved points
	Silicon	42.9	1,182	0.07	(100)	Face-centered cubic system
	Copper	28.2	778	0.05	(100)	Face-centered cubic system
	Cu_2_O	13.0	357	0.02	(100)	Primitive cubic system
Cu/PS/Si (100)	Not detected	41.9	1,436	0.08	None	Unsolved points
	Silicon	37.3	1,278	0.07	(100)	Face-centered cubic system
	Copper	20.3	695	0.04	(100)	Face-centered cubic system
	Cu_2_O	0.5	16	0.00	(100)	Primitive cubic system
Cu/Si (111)	Not detected	0.00	0	0.00	None	Unsolved points
	Silicon	64.3	2,140	0.12	(111)	Face-centered cubic system
	Copper	32.0	1,065	0.06	(111)	Face-centered cubic system
	Cu_2_O	3.8	125	0.01	(111)	Primitive cubic system
Cu/PS/Si (111)	Not detected	26.0	863	0.05	None	Unsolved points
	Silicon	49.5	1,642	0.10	(111)	Face-centered cubic system
	Copper	23.2	770	0.04	(111)	Face-centered cubic system
	Cu_2_O	1.3	42	0.00	(111)	Primitive cubic system

Cu was deposited for 4 s from 0.025 M CuSO4·5H2O + 0.005 M HF aqueous solution.

We suppose that the limited number of broken bonds of the Si (100) surface causes incomplete reduction of Cu^2+^ to Cu^+^ in some places. Thus, oxygen from the environment has an opportunity to give its electrons to Cu^+^ that is connected with the Si surface. Furthermore, correlation of such result with SEM allows us to conclude that the greater amount of Cu_2_O can be due to larger sizes of Cu particles.

**Figure 4:**
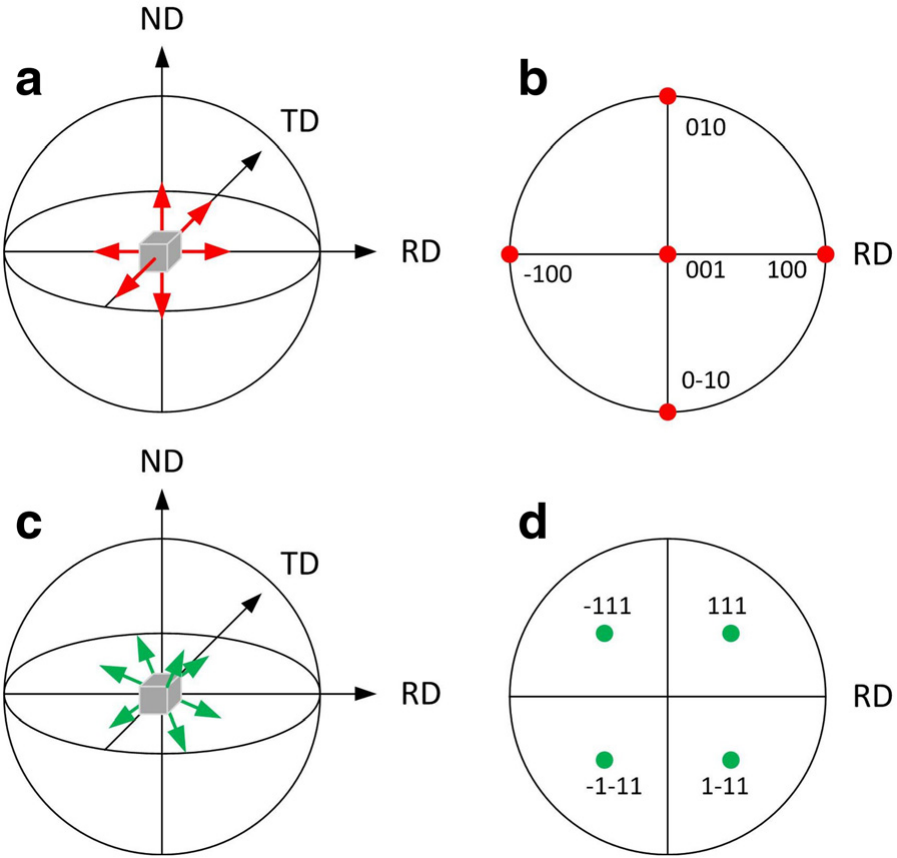
**Stereographic projections (pole maps) of a cubic unit cell orientation (001). **(**a**) Six (001) plane normal (poles) are shown, (**b**) stereographic projection of these directions which is a (100) pole figure of this crystal orientation, (**c**) eight (111) plane normals are shown, and (**d**) stereographic projection of these directions which is a (111) pole figure of this crystal orientation.

**Figure 5:**
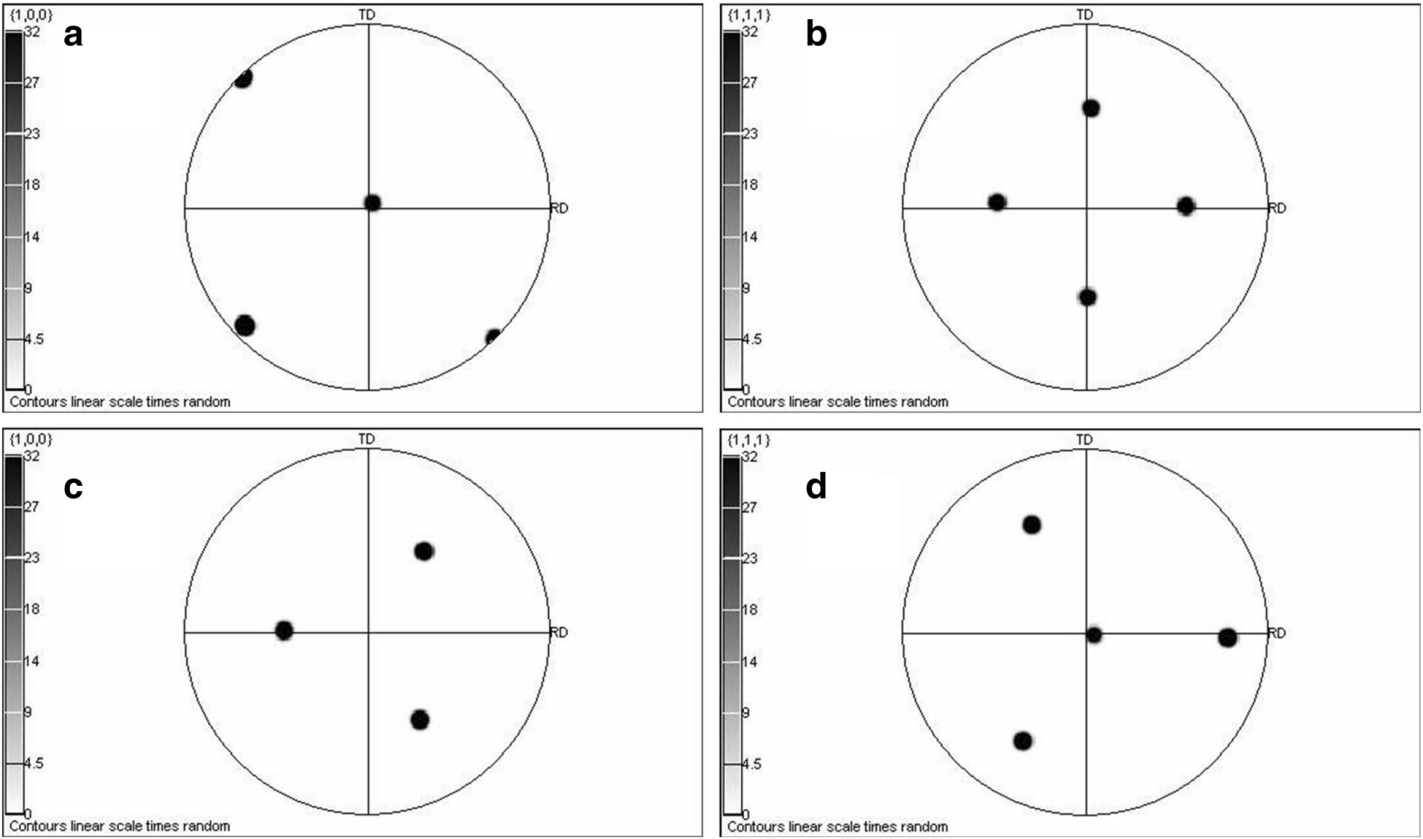
**EBSD pole maps.** Figures obtained by stereographic projection of the (**a**, **c**) [100] and (**b**, **d**) [111] crystallographic directions in the Si, Cu, Cu_2_O crystals of (a, b) Cu/Si (100) and Cu/PS/Si (100), (c, d) Cu/Si (111) and Cu/PS/Si (111) samples.

EBSD technique allows the revealing of orientation of the crystalline phase. It is provided by the stereographic projection of crystallographic directions, resulting in the creation of pole maps for the differently orientated crystals. Figure 4 presents the principle of the pole mapping where ND is for normal direction, TD is for transverse direction, and RD is for rolling direction. Figure 4a,c shows the reference spheres, and Figure 4b,d shows the projection planes. Thus (hkl) pole figure results in the distribution of the [hkl] directions in the sample. The pole maps obtained for all crystalline phases of the samples showed that Cu and Cu_2_O crystals grown on Si and PS inherited the orientation of the original Si substrate (Figure 5) although their lattice parameters are very different (*a*_Si_ = 0.5431 nm, *a*_Cu_ = 0.3615 nm).

### Open-circuit potential

**Figure 6:**
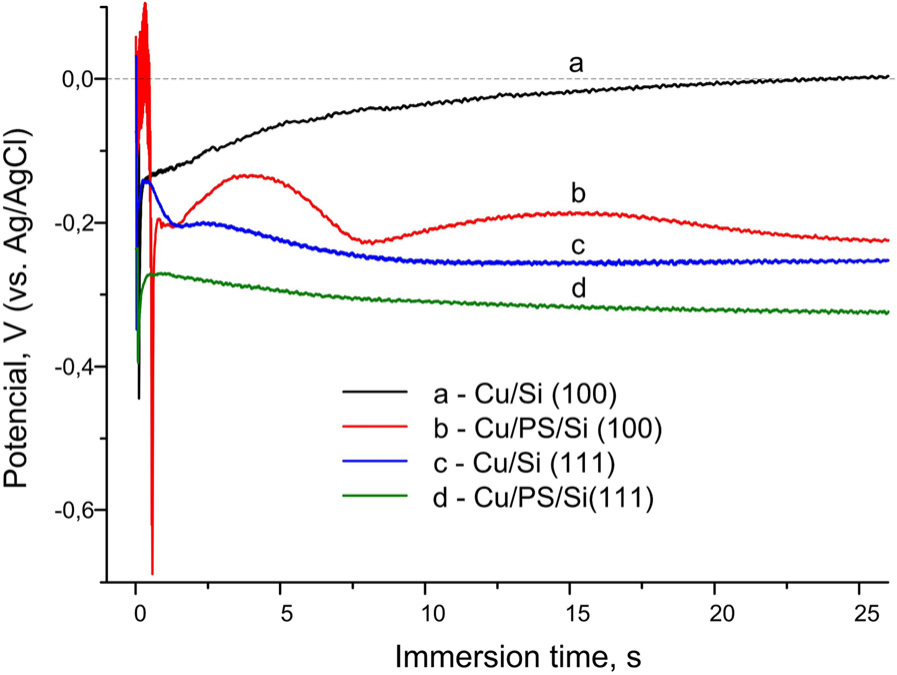
**OCP vs immersion time.** (curve a) Cu/Si (100), (curve b) Cu/PS/Si (100), (curve c) Cu/Si (111), and (curve d) Cu/PS/Si (111).

It is known that immersion deposition of metals on bulk Si and PS is accompanied by changes of the surface potential of the substrate which are connected with charge transfer due to Si atom oxidation and metal reduction [4]. That is why observation of OCP behavior allows the revelation of the regularities of immersion deposition. Figure 6 shows the time-dependent OCP responses of the bulk Si and PS samples of (111) and (100) orientations immersed into the solution for Cu deposition. The measurements were performed under normal room light at 25°C. The immersion moment of substrates into the solution was accompanied by a sharp decrease of the potential value related to surface destabilization. For the PS samples, these peaks are more negative than for the bulk Si of the corresponding orientation because of the breaking SiH_*x*_ bonds of the PS surface in the solution. The potentials then rose in the more positive direction since the adsorption and nucleation of Cu. Further growth of Cu particles resulted in the slight decrease of the potential for the samples based on PS/Si (100), Si (111), and PS/Si (111). Several peaks of the OCP time dependencies have to be related to the periodical coalescence of Cu particles during immersion deposition [10]. It is seen that Si (100) OCP demonstrates different behaviors than of the other samples. It gradually increased without any peaking. The sizes of Cu particles on the bulk Si (100) were larger than those on the other samples, and their density was significantly less, which means that more surface area of Si in contrast with bulk Si (111) and PS samples was opened for the permanent adherence and nucleation of Cu. That is why the potential constantly rose. Moreover, the potential of Si (100) overcame the 0 value at 23 s of the Cu immersion deposition and shifted to the positive direction. At the same time, the potential of the other samples is always shifted to the negative direction and does not cross the 0 value. This effect has been already explained and addressed to the negative potential at which Cu^0^ is stable, while the positive potential shift means stability of Cu^+^ or Cu^2+^[18,25]. Thus, the potential shift to the positive value can take a place in cases of incomplete Cu reduction or dissolution of the deposited Cu [25]. As we have observed the greatest amount of Cu_2_O for the bulk Si (100) sample, the incomplete reduction of the adsorbed Cu ions is more likely to happen.

## Conclusions

We studied the initial stages of Cu immersion deposition from the aqueous solution of Cu sulfate in the presence of hydrofluoric acid on bulk and porous silicon. The analysis of top-view SEM images of the samples revealed that Cu deposited both on the bulk and porous silicon as a layer of NPs in accordance with the Volmer-Weber mechanism. The size distribution of Cu NPs for all samples had a bimodal character and a minimum peak between 40 and 50 nm. The Si (100) substrate allowed the depositing of Cu particles of the largest sizes that reached the range of 200 to 210 nm. The smallest Cu NPs were detected on Si (111). The densities of Cu NPs on Si (100) and Si (111) differed greatly and were 10^9^ and 10^10^ cm^−2^, respectively. At the same time, the PS substrates resulted in the almost equal sizes and densities of Cu NPs. EBSD analysis showed that Cu NPs grew as crystals with a maximum size of 10 nm and inherited the orientation of the original silicon substrate. We suppose that this fact partially promotes the improvement of thick metal films' adhesion to Si substrates previously covered with Cu/PS layer [11,12]. In addition, EBSD detected crystals of Cu_2_O on all samples, but Cu NPs on Si (100) were the most oxidized. Moreover, Cu deposited on the porous substrates demonstrated greater stability to the oxidation in contrast with bulk Si. Consequently, the crystal orientation of the original Si wafer significantly affected the sizes, density, and oxidation level of Cu NPs deposited by immersion technique only on bulk Si in contrast to PS.

The possibility to control the structural parameters and oxidation stability of Cu NPs on bulk and porous Si can allow the improvement of the adhesion and conductive characteristics of metal interconnections. We suppose as well that the revealed regularities of Cu immersion deposition are valid for the other metals of cubic lattice cell.

## Abbreviations

EBSD: Electron backscatter diffraction;NCs: Nanocomposites;NPs: Nanoparticles;PS: Porous silicon;OCP: Open-circuit potential;SEM: Scanning electron microscopy

## Competing interests

The authors declare that they have no competing interests.

## Authors’ contributions

HB carried out the fabrication of samples and gravimetric and OCP measurements, designed, and drafted the manuscript. SLP, RF, and AV performed and explained the EBSD analysis. PN carried out the SEM and its quantification. MB and VB initiated, planned, and controlled the research process. All authors read and approved the final manuscript.
